# In relation to NO-System, Stable Pentadecapeptide BPC 157 Counteracts Lidocaine-Induced Adverse Effects in Rats and Depolarisation In Vitro

**DOI:** 10.1155/2020/6805354

**Published:** 2020-05-27

**Authors:** Marin Lozic, Vasilije Stambolija, Ivan Krezic, Aleksandra Dugandzic, Gordana Zivanovic-Posilovic, Slaven Gojkovic, Josip Kovacevic, Luka Vrdoljak, Ivan Mirkovic, Antonio Kokot, Andreja Petrovic, Katarina Horvat Pavlov, Domagoj Drmic, Jelena Suran, Alenka Boban Blagaic, Sven Seiwerth, Predrag Sikiric

**Affiliations:** Department of Pharmacology, School of Medicine, Medical Faculty, University of Zagreb, Salata 11, P.O. Box 916, 10000 Zagreb, Croatia

## Abstract

Recently, the pentadecapeptide BPC 157-induced counteraction of bupivacaine cardiotoxicity has been reported. Medication includes (i) lidocaine-induced local anesthesia via intraplantar application and axillary and spinal (L4-L5) intrathecal block, (ii) lidocaine-induced arrhythmias, (iii) convulsions, and (iv) lidocaine-induced HEK293 cell depolarisation. BPC 157 applications (intraplantar, intraperitoneal, and intragastric) were given (i) immediately after lidocaine, (ii) 10 min after, or (iii) 5 min before. The BPC 157/NO-system relationship was verified with NO-agents, the NOS-blocker L-NAME and the NOS-substrate L-arginine, given alone and/or together, in axillary and spinal intrathecal blocks. BPC 157 applied immediately after lidocaine or 5 min before the application of lidocaine considerably ameliorated plantar presentation. BPC 157 medication considerably counteracted lidocaine-induced limb function failure; L-NAME was counteracted; L-arginine exhibited counteraction when given immediately after lidocaine, but prolongation was seen when given later. Given together, prophylactically or therapeutically, L-NAME and L-arginine (L-NAME + L-arginine) counteracted the other's response. BPC 157 maintained its original response when given together with L-NAME or L-arginine. When BPC 157 was given together with L-NAME and L-arginine, its original response reappeared. BPC 157 antagonised the lidocaine-induced bradycardia and eliminated tonic-clonic convulsions. Also, BPC 157 counteracted the lidocaine-induced depolarisation of HEK293 cells. Thus, BPC 157 has antidote activity in its own right against lidocaine and local anesthetics.

## 1. Introduction

We focused on the counteracting relationship between the stable gastric pentadecapeptide BPC 157 [1–13] and lidocaine, local anesthesia, infiltrative cutaneous analgesia, axillary and spinal intrathecal blocks, NO-system [1–13], lidocaine-induced arrhythmias, convulsions, and lidocaine-induced depolarisation in HEK293 cells.

Specific arguments to extend the use of BPC 157 against the adverse effects of local anesthetics, such as lidocaine, follow the BPC 157 counteraction of bupivacaine cardiotoxicity [14], bupivacaine-induced depolarisation in HEK293 cells [14], and drug-induced heart arrhythmias [15–19]. A similar generalization was already made with dopamine neuroleptics or prokinetics since BPC 157 counteracts the prolongation of the QTc interval after the daily administration of dopamine neuroleptics or prokinetics [19] much like neuroleptic-induced catalepsy and gastric ulcers [19–22]. Likely, a particular extended BPC 157's beneficial effect may combine the antagonism of the entire spectrum of local anesthetic-induced neurotoxic and cardiotoxic effects.

In addition, BPC 157 interacts with peptidergic sensory afferent neurons and may rescue adult and newborn capsaicin rats [23, 24], improves the healing of damaged enteric nerves, and increases the survival of cultured enteric neurons and the proliferation of cultured enteric glial cells [25]. Also, BPC 157 attenuated morphine analgesia and counteracted the haloperidol-induced enhancement of the antinociceptive action of morphine [26]. Also, BPC 157 counteracts the adverse effects of NSAIDs, both COX1 and COX2 inhibitors [7, 24, 27–34]. BPC 157 markedly improved rat sciatic nerve healing following nerve transection and/or anastomosis [35]. After an induced traumatic brain injury, there is a marked attenuation of damage with an improved early outcome and minimal postponed mortality throughout a 24 h postinjury period with less intense subarachnoid and intraventricular haemorrhage and brain laceration and subsequent brain oedema considerably improved [36]. Also, BPC 157 counteracts encephalopathies after NSAID treatment [7, 27–29, 31, 32], insulin overdose [37], and a multiple sclerosis rat model induced by neurotoxin cuprizone application [38]. Finally, BPC 157 counteracts various induced seizures in rats and mice [27, 37, 39]. As an original antiulcer peptide, BPC 157 has virtually no known toxicity of its own, an LD1 value has not yet been reported, and there have been no side effects in clinical trials, such as ulcerative colitis and multiple sclerosis [1–13].

Also, BPC 157 largely interacts with NO-system and may counteract the adverse effect of NO-synthase-blockade (i.e., L-NAME) much like the adverse effect of NOS overstimulation (L-arginine). In the significant antagonism of general anesthesia produced by intravenous general anesthetic thiopental, BPC 157 caused a parallel shift of the dose-response curve to the right and abolished the potentiating effects of L-NAME [40].

On the other hand, BPC 157 produced analgesia in the MgSO_4_ and acetic acid test in mice, a model of prolonged pain associated with tissue injury [41]. This indicates that BPC 157 may have local anesthetic activity on its own.

Thus, we attempt to counteract lidocaine toxicity. Previously reported effective dose regimens of the pentadecapeptide BPC 157 [14–19], along with the NOS-inhibitor L-NAME and the NOS-substrate L-arginine, given alone and/or in combination, were administered after lidocaine application in rats or in an in vitro HEK293 cell model, showing the presence of endogenous voltage-gated potassium (K^+^), sodium (Na^+^), and calcium (Ca^2+^) channels [42].

## 2. Materials and Methods

### 2.1. Animals

Study protocols were conducted in male albino Wistar rats, with a body weight of 200–300 g; animals were randomly assigned and used in all of the experiments, with 6 rats/group/interval. The protocol was approved by the local Ethics Committee (case number 380-59-10106-17-100/290) and by the Directorate of Veterinary science (UP/I-322-01/15-01/22). They were in-house bred by the Pharmacology Animal Facility at the School of Medicine, Zagreb, Croatia. The animal facility is registered by the Directorate of Veterinary Science under Reg. no. HR-POK-007. Laboratory rats were acclimated for 5 days and randomly assigned to their respective treatment groups. Laboratory animals were housed in PC cages in conventional laboratory conditions at the temperature of 20–24°C, relative humidity of 40–70%, and noise level of 60 dB. Each cage was identified by the following: number of the study, group, dose, and number and sex of each animal. Fluorescent lighting provided illumination 12 hours per day. Standard GLP diet and fresh water were provided ad libitum. Animal care was in compliance with the SOPs of the Pharmacology Animal Facility and the European Convention for the protection of vertebrate animals used for experimental and other scientific purposes (ETS 123).

Ethical principles of the study ensured compliance with the European Directive 010/63/E, the Law on Amendments to Animal Protection Act (Official Gazette 32/19, the Animal Protection Act (Official Gazette 102/17), Ordinance on the protection of animals used for scientific purposes (Official Gazette 55/13), FELASA recommendations, and recommendations of the Ethics Committee of the School of Medicine, University of Zagreb. Experiments were assessed by observers who were unaware of the given treatment.

### 2.2. Drugs

As previously mentioned, without a carrier or peptidase inhibitor, stable gastric pentadecapeptide BPC 157 (a partial sequence of the human gastric juice protein BPC, freely soluble in water at pH 7.0 and in saline) was included [1–13]. It was prepared as a peptide with 99% (HPLC) purity (1-des-Gly peptide was the main impurity; manufactured by Diagen, Ljubljana, Slovenia, GEPPPGKPADDAGLV, M.W. 1419) in dose and application regimens, as described previously [1–13]. Lidocaine (Lidokain, 2% Belupo, Croatia), L-NAME and L-arginine (Sigma Aldrich) were commercially purchased and used accordingly [1–13].

### 2.3. Lidocaine-Induced Arrhythmias, Local Anesthesia, and Convulsions Effects

#### 2.3.1. Lidocaine-Induced Arrhythmias

To induce arrhythmias, we applied lidocaine (80 mg/kg) intraperitoneally. Medication (BPC 157 10 *μ*g/kg, 10 ng/kg intraperitoneally) was applied 30 min before lidocaine or immediately after lidocaine, while the control animals received a simultaneous corresponding volume of saline (5 ml/kg intraperitoneally). As described previously, the ECG was recorded continuously for 60 minutes [15, 16] using all three main leads by positioning stainless steel electrodes on all four limbs; an ECG monitor (Medtronic Programmer, USA) connected to a digital oscilloscope (LeCroy Waverunner LT342, USA) was used, which enabled precise recording, measurement, and analysis of ECG parameters, as described in detail previously.

#### 2.3.2. Lidocaine-Induced Local Anesthesia

(1) Hot plate test: for infiltrative cutaneous anesthesia, an intraplantar application of lidocaine (4 mg/kg) was applied in each foot (0.1 ml/foot) 3 minutes before rats were placed on a hot plate with a thermostatic base maintaining its mean temperature at 55 ± 0.5°C using a previously applied procedure [26]. Time (seconds) to the onset of the rat licking or lifting its hind paws or jumping (whichever occurred first) was recorded. To prevent greater tissue damage, the time spent by the rat on the hot plate was limited to 120 seconds (cutoff time), and the immediate extent of the plantar damage was scored (0–3) as follows: 0, presentation without oedema; 1, moderate oedema; 2, mild oedema; 3, severe oedema. Medication (BPC 157 10 *μ*g/kg, 10 ng/kg) was applied immediately after lidocaine was given as an intraplantar application in each foot (0.1 mL/foot) or via intraperitoneal or intragastric application (5 ml/kg) 5 min before intraperitoneal lidocaine, while controls received a corresponding volume of saline.

(2) Axillary block: perineural anesthesia of the nerves of the left brachial plexus was performed with lidocaine (0.3 ml, 6 mg/kg) in isoflurane inhalation lightly anesthetized rats and scored accordingly (0–5) (0, forelimb plegia without muscle tone and unable to walk or grasp the wire mesh; 1, forelimb in flexion and adduction, weak use of the forearm during tottering and walking, and unable to grasp the wire mesh; 2, while walking, use of the forelimb paw was directed medially and inverted, digits showed contracture, and unable to grasp the wire mesh; 3, normal walking, unable to grasp the wire mesh, and maintained grasping for 5 seconds when put in a vertical position; 4, normal walking, able to grasp the wire mesh, and maintained grasping for 10 seconds when put in a vertical position; 5, normal walking, able to grasp the wire mesh, and maintained grasping for more than 10 seconds when put in a vertical position); the immediate extent of paw damage was also scored (0–3) (0, presentation without oedema; 1, moderate oedema; 2, mild oedema; 3, severe oedema) in 5-minute intervals during the next 125 minutes. Medication (BPC 157 10 *μ*g/kg, 10 ng/kg intraperitoneally and L-NAME (5 mg/kg) and/or L-arginine (100 mg/kg) intraperitoneally, alone and/or together) was applied immediately after lidocaine or 10 minutes after lidocaine while the controls simultaneously received a corresponding volume of saline.

(3) Spinal block: intrathecal anesthesia was performed at the L4-L5 level of the rat spine [43]. Using the 27-gauge needle and a 1 cc syringe, we applied lidocaine (0.1 mL, 4 mg/kg) by entering the subarachnoid spinal space. Successful entry into the intrathecal space of the spinal cord of the rats was confirmed by the flick of the tail and/or the lower limbs and then lidocaine was applied intrathecally. The assessment of the spontaneous movement of the legs and feet was performed by using the modified Bromage scale [44] (0, nil motor deficit; 1, just able to flex the knees with free movement of the feet, partial motor deficit (33%); 2, unable to flex knees, but free movement of feet, almost complete motor deficit (66%); 3, unable to move legs or feet, complete motor deficit (100%)) in 5-minute intervals during the next 125 minutes. Medication (BPC 157 10 *μ*g/kg, 10 ng/kg intraperitoneally or intragastrically and L-NAME (5 mg/kg) and/or L-arginine (100 mg/kg) intraperitoneally, alone and/or together) was applied immediately after lidocaine or 10 minutes after lidocaine, while controls simultaneously received a corresponding volume of saline.

#### 2.3.3. Lidocaine-Induced Seizures

To induce seizures, we applied lidocaine (80 mg/kg) intraperitoneally. Medication (BPC 157 10 *μ*g/kg, 10 ng/kg intraperitoneally) was immediately given after lidocaine, while the controls simultaneously received a corresponding volume of saline (5 ml/kg intraperitoneally). Assessment included time (minutes) to onset and the end of seizures in the rats and the number of rats with seizures.

#### 2.3.4. Patch-Clamp Studies

Coverslips with HEK293 cells were mounted at the bottom of a perfusion chamber on an inverted microscope (Axiovert 10, Zeiss, Germany). Membrane voltages (Vm) of HEK293 cells were measured using the slow-whole-cell patch-clamp technique [15, 45]. A Ringer's-type solution containing 145 mM·NaCl, 1.6 mM·K_2_HPO_4_, 0.4 mM·KH_2_PO_4_, 5 mM·D-glucose, 1 mM·MgCl_2_, and 1.3 mM·calcium gluconate (pH 7.4) was utilised. Lidocaine (1 mM) and BPC 157 (1 *μ*m) were dissolved in this solution. All of the experiments were performed at 37°C with a bath perfusion rate of 10 ml/min. Patch-clamp pipettes were filled with 95 mM·potassium gluconate, 30 mM·KCl, 4.8 mM·Na_2_HPO_4_, 1.2 mM·NaH_2_PO_4_, 5 mM·D-glucose, 1.3 mM·calcium gluconate, 1.03 mM·MgCl_2_, and 1 mM·ATP (pH 7.2). To this solution, 160 *μ*m nystatin was added to permeabilise the membrane using the pipette. The pipette resistance was 5–10 MΩ. Vm was measured with a patch-clamp amplifier (U. Fröbe Physiologische Institut, Germany) and recorded continuously on a pen recorder (WeKa graph, Switzerland).

### 2.4. Statistical Analysis

Statistical analysis was performed by parametric one-way ANOVA with the post hoc Newman–Keuls test and the nonparametric Kruskal–Wallis and subsequent Mann–Whitney *U* test to compare groups. Values are presented as mean ± SD and as minimum/median/maximum. To compare the difference in frequency between the groups, the chi-square test or Fischer's exact test was used. *P* < 0.05 was considered statistically significant.

## 3. Results

The results obtained show that the beneficial effect of BPC 157 may combine the antagonism of the lidocaine-induced arrhythmias, local anesthesia, and convulsions effects (Figure 1–11) in rats, with the lidocaine-induced depolarisation in HEK293 cells (Figure 12).

### 3.1. Lidocaine-Induced Arrhythmias

**Figure 1:**
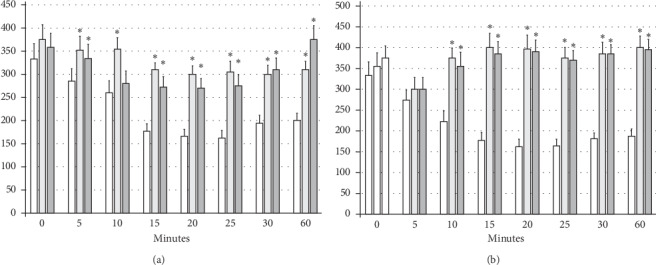
Bradycardia: the intraperitoneal application of lidocaine (80 mg/kg) induced significant bradycardia within minutes (white bars) which BPC 157 opposed (gray bars). Prophylactic effect: the application of BPC 157 (10 *μ*g/kg (light gray bars) or 10 ng/kg (dark gray bars)) counteracted the development of lidocaine-induced bradycardia (given 30 minutes before the application of lidocaine) (a). Therapy effect: BPC 157 (10 *μ*g/kg (light gray bars) or 10 ng/kg (dark gray bars)) reversed (given immediately after lidocaine) the already established lidocaine-induced bradycardia) (b). ^*∗*^*P* < 0.05 at least vs. control. Heart frequency, beats/min; values are presented as mean ± SD.

The intraperitoneal application of lidocaine (80 mg/kg) induced significant bradycardia within minutes (Figure 1). The application of BPC 157 (10 *μ*g/kg or 10 *μ*g/kg) counteracted the development of lidocaine-induced bradycardia (given 30 minutes before the application of lidocaine) and reversed (given immediately after lidocaine) the already established lidocaine-induced bradycardia (Figure 1).

### 3.2. Lidocaine-Induced Local Anesthesia

#### 3.2.1. Hot Plate Test

**Figure 2:**
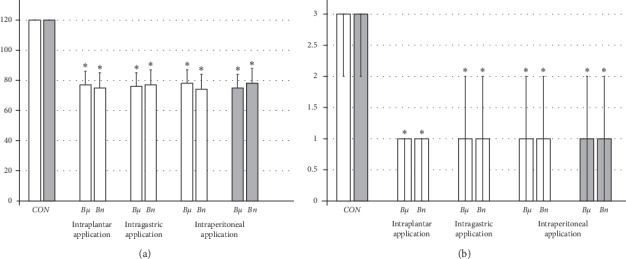
Hot plate test: lidocaine intraplantar (4 mg/kg, 0.1 ml/foot) application produced complete anesthesia (left) and considerable plantar damage (right) (*CON*). Therapy effect: BPC 157 medication (10 *μ*g/kg (*Bμ*), 10 ng/kg (*Bn*)) applied immediately after lidocaine (white bars), as an intraplantar , intraperitoneal, or intragastric application in each foot, considerably counteracted local anesthesia and ameliorated posthot plate test-induced plantar presentation (middle). Prophylactic effect: BPC 157 medication (10 *μ*g/kg (*Bμ*), 10 ng/kg (*Bn*)) applied intraperitoneally at 5 min before lidocaine (gray bars) considerably counteracted local anesthesia and ameliorated posthot plate test-induced plantar presentation (right). (a) Hot plate test, time with no pain reaction, sec. (b) Hot plate test-induced plantar oedema, scored 0–3; values are presented as mean ± SD and min/med/max. ^*∗*^*P* < 0.05 at least vs. control.

The intraplantar application of lidocaine in each foot (4 mg/kg, 0.1 ml/foot) produced complete anesthesia during the hot plate test, with no changes in pain reaction (absent licking or lifting hind paws or jumping) during the entire observation period (120 seconds); this led to severe (score 3) paw oedema (Figures 2 and 11). On the other hand, BPC 157 medication (10 *μ*g/kg or 10 ng/kg) applied immediately after or 5 min before lidocaine, regardless of the mode of administration (intraplantar, intraperitoneal, or intragastric application in each foot), considerably decreased the time of lidocaine-induced analgesia, with moderate to mild oedema, and ameliorated posthot plate test-induced plantar presentation (Figures 2 and 11).

#### 3.2.2. Axillary Block

**Figure 3:**
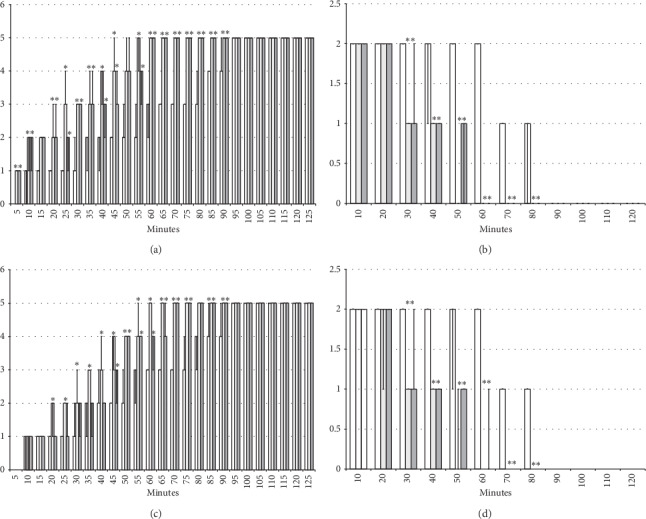
Axillary block: left brachial plexus perineural anesthesia produced considerable and long-lasting function failure of the affected fore limb and an inability to walk and to grasp (left) along with considerable fore limb oedema in the anesthetized rats (right) (white bars) which BPC 157 opposed (gray bars). Prophylactic effect: BPC 157 medication (gray bars) (10 *μ*g/kg (light gray bars), 10 ng/kg (dark gray bars) intraperitoneally) given immediately after lidocaine (upper) considerably counteracted lidocaine-induced course. Therapy effect: BPC 157 medication (gray bars) (10 *μ*g/kg (light gray bars), 10 ng/kg (dark gray bars) intraperitoneally) given later (after 10 minutes) (lower) considerably counteracted lidocaine-induced course. (a), (b) Perineural anesthesia of the nerves of the left brachial plexus, scored 0–5, min/med/max. (c), (d) Fore limb oedema with perineural anesthesia of the nerves of the left brachial plexus, scored 0–3; values are presented as min/med/max. ^*∗*^*P* < 0.05 at least vs. *control*.

**Figure 4:**
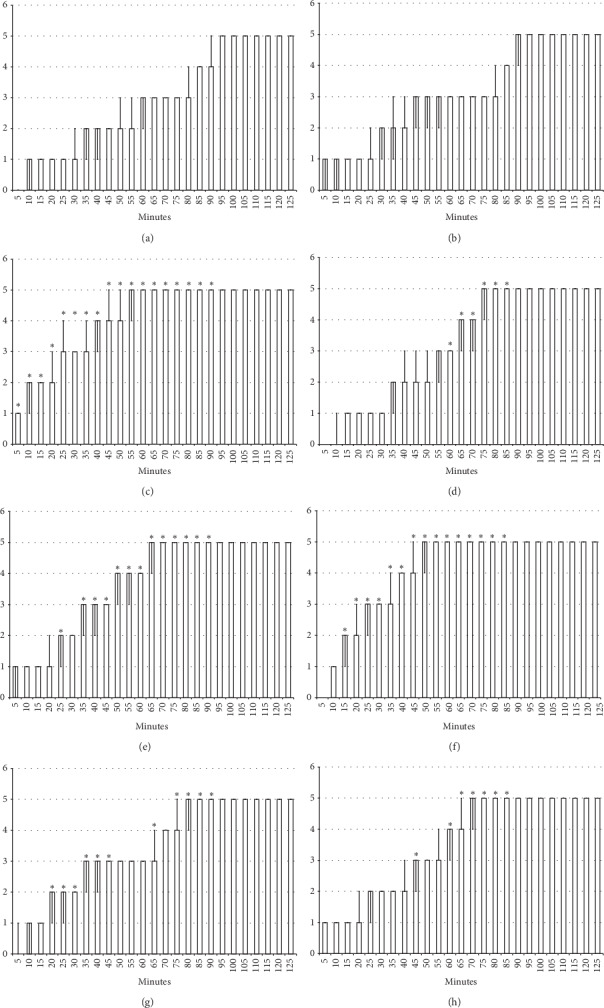
Axillary block (function failure): left brachial plexus perineural anesthesia (white bars) produced considerable and long-lasting function failure of the affected fore limb and an inability to walk and to grasp. The prophylactic effect of the agents given immediately after lidocaine, saline 5 ml/kg, BPC 157 10 *μ*g/kg, L-NAME 5 mg/kg, and L-arginine 100 mg/kg intraperitoneally, alone and/or together. Perineural anesthesia of the nerves of the left brachial plexus, scored 0–5. (a) Saline (control). (b) L-NAME + L-arginine. (c) BPC 157. (d) L-arginine + BPC 157. (e) L-NAME. (f) L-NAME + BPC 157. (g) L-arginine. (h) L-NAME + L-arginine + BPC 157. Values are presented as min/med/max. ^*∗*^*P* < 0.05 at least vs. control.

**Figure 5:**
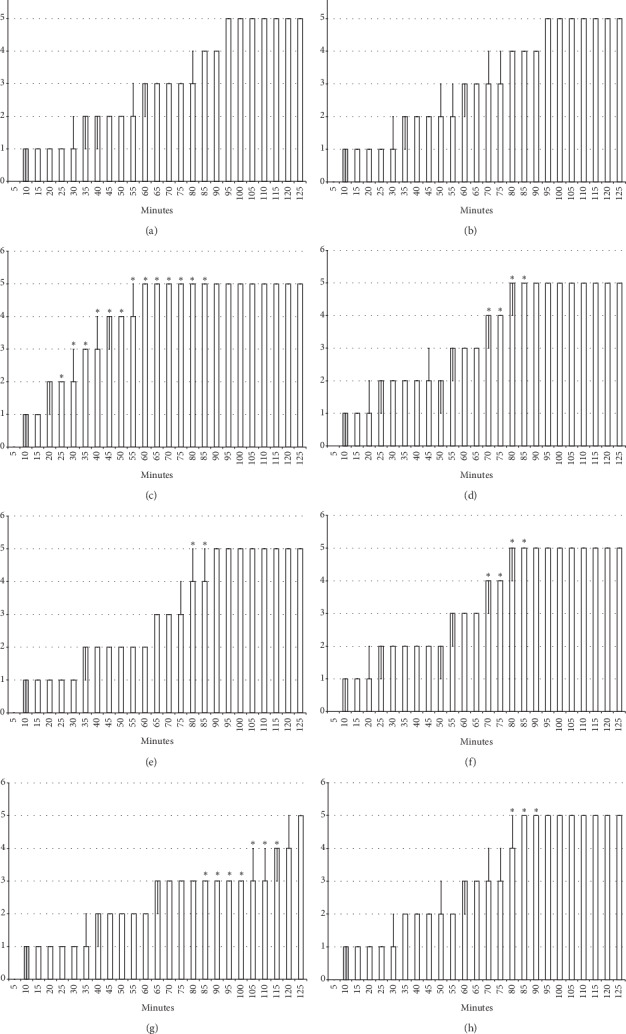
Axillary block (function failure): left brachial plexus perineural anesthesia (white bars) produced considerable and long-lasting function failure of the affected fore limb and an inability to walk and to grasp. Therapy effect of the agents (saline 5 ml/kg, BPC 157 10 *μ*g/kg, L-NAME 5 mg/kg, and L-arginine 100 mg/kg intraperitoneally, alone and/or together) given later at 10 minutes after lidocaine (gray bars indicate presentation at 10 min lidocaine-time). Perineural anesthesia of the nerves of the left brachial plexus, scored 0–5. (a) Saline (control). (b) L-NAME + L-arginine. (c) BPC 157. (d) L-NAME + BPC 157. (e) L-NAME. (f) L-NAME + BPC 157. (g) L-arginine. (h) L-NAME + L-arginine + BPC 157. Values are presented as min/med/max. ^*∗*^*P* < 0.05 at least vs. control.

**Figure 6:**
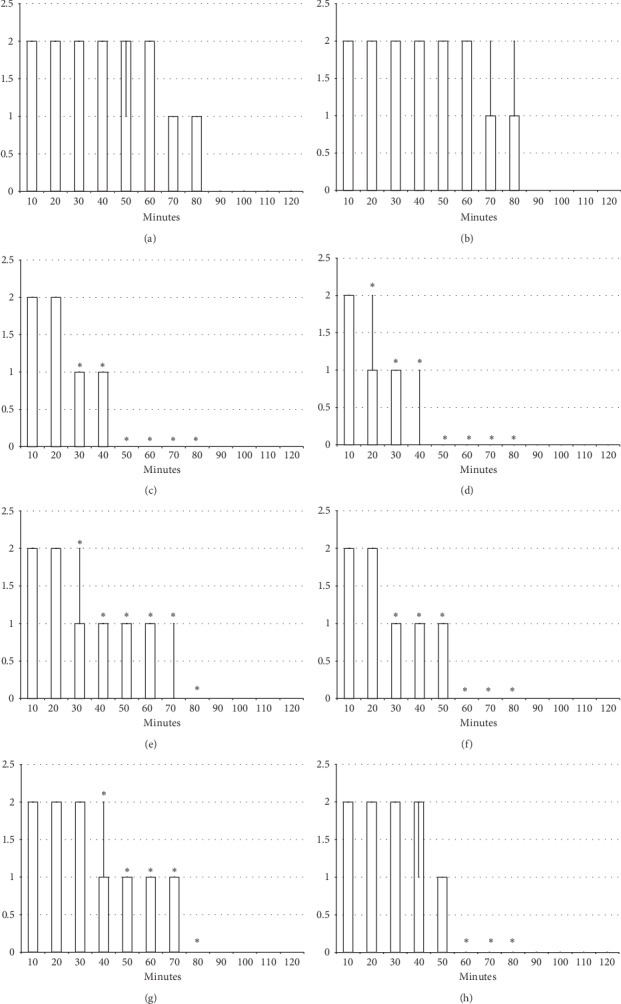
Axillary block (limb oedema): left brachial plexus perineural anesthesia (white bars) produced considerable fore limb oedema in the anesthetized rats. The prophylactic effect of the agents given immediately after lidocaine, saline 5 ml/kg, BPC 157 10 *μ*g/kg, L-NAME 5 mg/kg, and L-arginine 100 mg/kg intraperitoneally, alone and/or together. Fore limb oedema with perineural anesthesia of the nerves of the left brachial plexus, scored 0–3. (a) Saline (control). (b) L-NAME + L-arginine. (c) BPC 157. (d) L-arginine + BPC 157. (e) L-NAME. (f) L-NAME + BPC 157. (g) L-arginine. (h) L-NAME + L-arginine + BPC 157. Values are presented as min/med/max. ^*∗*^*P* < 0.05 at least vs. control.

**Figure 7:**
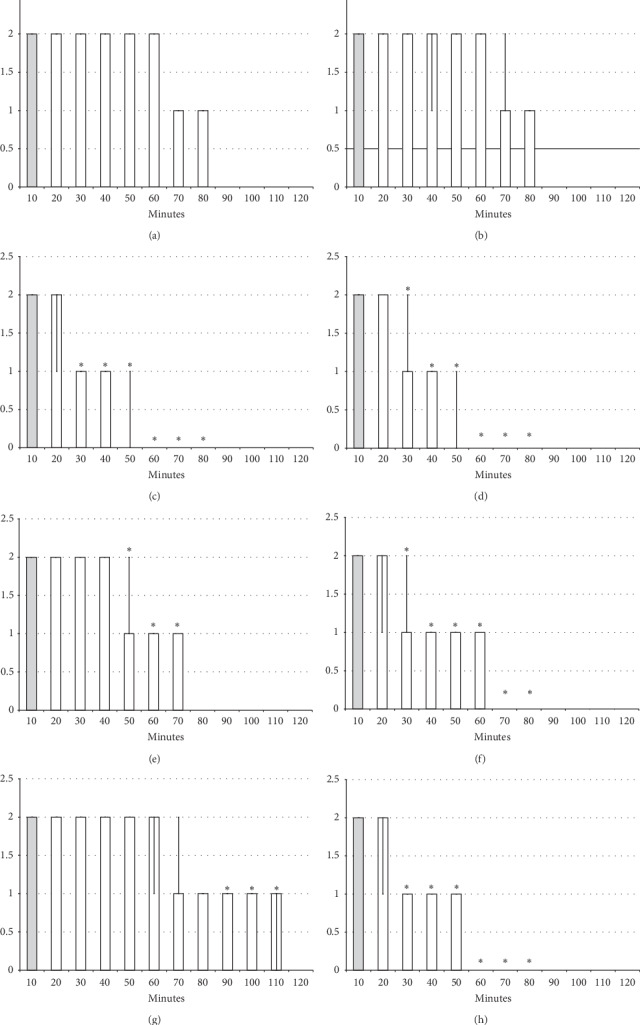
Axillary block (limb oedema): left brachial plexus perineural anesthesia (white bars) produced considerable fore limb oedema in the anesthetized rats. Therapy effect of the agents (saline 5 ml/kg, BPC 157 10 *μ*g/kg, L-NAME 5 mg/kg, and L-arginine 100 mg/kg intraperitoneally, alone and/or together) given later at 10 minutes after lidocaine (gray bars indicate presentation at 10 min lidocaine-time). Fore limb oedema with perineural anesthesia of the nerves of the left brachial plexus, scored 0–3. (a) Saline (control). (b) L-NAME + L-arginine. (c) BPC 157. (d) L-arginine + BPC 157. (e) L-NAME. (f) L-NAME + BPC 157. (g) L-arginine. (h) L-NAME + L-arginine + BPC 157. Values are presented as min/med/max. ^*∗*^*P* < 0.05 at least vs. control.

Left brachial plexus perineural anesthesia produced considerable and long-lasting function failure of the affected fore limb and an inability to walk and grasp; complete recovery of the motor function of the affected limb (score 5) was only present after 90 minutes has passed since the application of lidocaine. The affected fore limb oedema was still present 90 minutes after lidocaine application (Figures 3–7 and 11). Given immediately after lidocaine, or 10 minutes later, BPC 157 medication in all dose regimes (10 *μ*g/kg or 10 ng/kg) counteracted lidocaine-induced axillary block with full recovery of the motor function presented approximately 60 minutes later and moderate affected limb oedema was withdrawn 50 minutes after lidocaine application. The application of L-NAME or L-arginine exhibited counteraction when given immediately after the application of lidocaine, with full limb motor function restored 70 minutes after lidocaine application. Given 10 minutes after lidocaine, L-NAME retained its effect; on the contrary, L-arginine application, 10 minutes after lidocaine prolonged anesthesia with full motor function reached not before the end of the observation period. Given together, prophylactically or therapeutically, L-NAME and L-arginine (L-NAME + L-arginine) counteracted each other's response; the results were similar to those of the control group. BPC 157 maintained its original response when given together with L-NAME and/or with L-arginine. Similar results were observed for oedema assessment (Figures 3–7 and 11).

#### 3.2.3. Spinal Block

**Figure 8:**
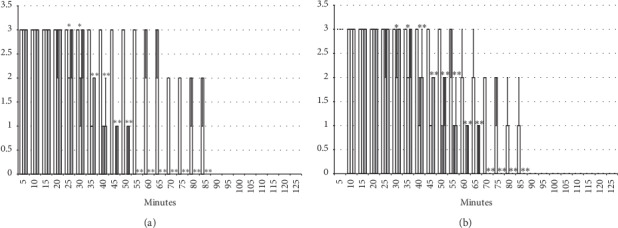
Spinal block (function failure and flaccid paralysis): intrathecal anesthesia produced a prolonged hind limbs function failure and long-lasting flaccid paralysis (white bars) which BPC 157 opposed. Prophylactic effect: BPC 157 medication (gray bars) (10 *μ*g/kg (light gray bars), 10 ng/kg (dark gray bars) intraperitoneally) given immediately after lidocaine (a) considerably counteracted lidocaine-induced course. Therapy effect: BPC 157 medication (gray bars) (10 *μ*g/kg (light gray bars), 10 *μ*g/kg (dark gray bars) intraperitoneally) given later, at 10 minutes after lidocaine (b), considerably counteracted lidocaine-induced course. With BPC 157 medication (10 *μ*g/kg, 10 *μ*g/kg intragastrically), an alike counteraction appears as well (data not specifically shown). Intrathecal anesthesia at the L4-L5 level rat spine, scored 0–3. Values are presented as min/med/max. ^*∗*^*P* < 0.05 at least vs. control.

**Figure 9:**
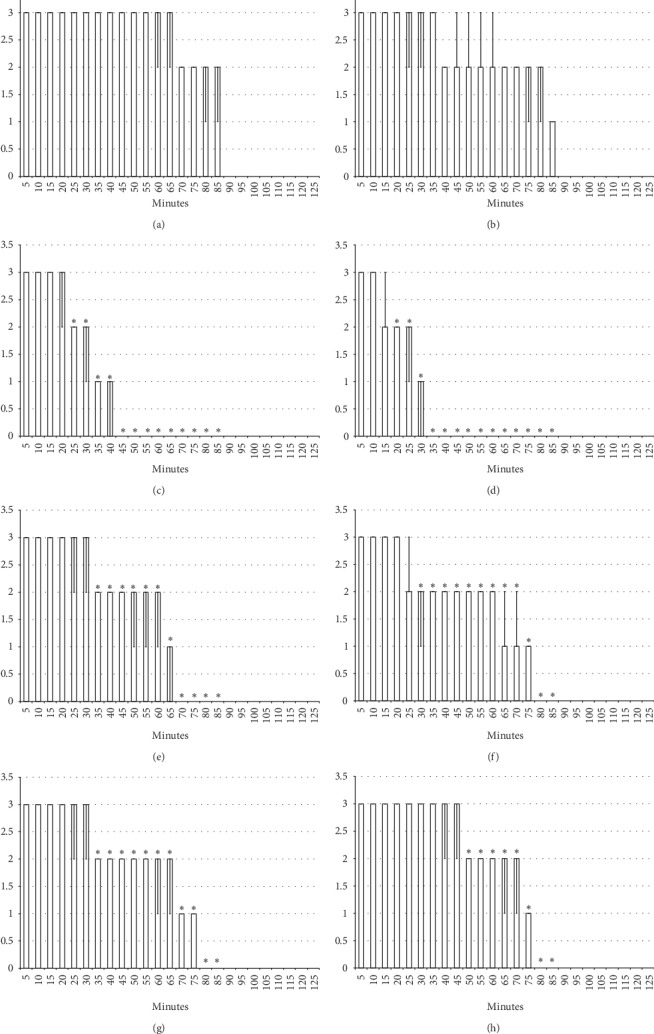
Spinal block (function failure and flaccid paralysis): intrathecal anesthesia prolonged hind limbs function failure and long-lasting flaccid paralysis (white bars). The prophylactic effect of the agents given immediately after lidocaine, saline 5 ml/kg, BPC 157 10 *μ*g/kg, L-NAME 5 mg/kg, and L-arginine 100 mg/kg intraperitoneally, alone and/or together. Intrathecal anesthesia at the L4-L5 level rat spine, scored 0–3. (a) Saline (control). (b) L-NAME + L-arginine. (c) BPC 157. (d) L-arginine + BPC 157. (e) L-NAME. (f) L-NAME + BPC 157. (g) L-arginine. (h) L-NAME + L-arginine + BPC 157. Values are presented as min/med/max. ^*∗*^*P* < 0.05 at least vs. control.

**Figure 10:**
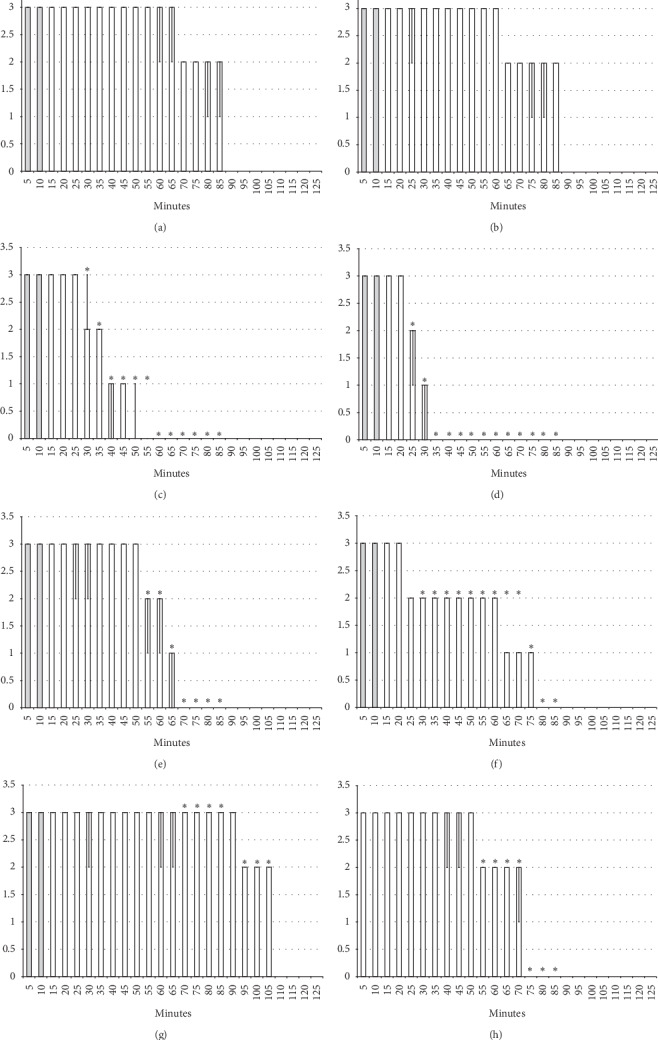
Spinal block (function failure and flaccid paralysis): intrathecal anesthesia prolonged hind limbs function failure and long-lasting flaccid paralysis (white bars). Therapy effect of the agents given later after lidocaine after 10 minutes (gray bars), saline 5 ml/kg, BPC 157 10 *μ*g/kg, L-NAME 5 mg/kg, and L-arginine 100 mg/kg intraperitoneally, alone and/or together. Intrathecal anesthesia at the L4-L5 level rat spine, scored 0–3. (a) Saline (control). (b) L-NAME + L-arginine. (c) BPC 157. (d) L-arginine + BPC 157. (e) L-NAME. (f) L-NAME + BPC 157. (g) L-arginine. (h) L-NAME + L-arginine + BPC 157. Values are presented as min/med/max. ^*∗*^*P* < 0.05 at least vs. control.

**Figure 11:**
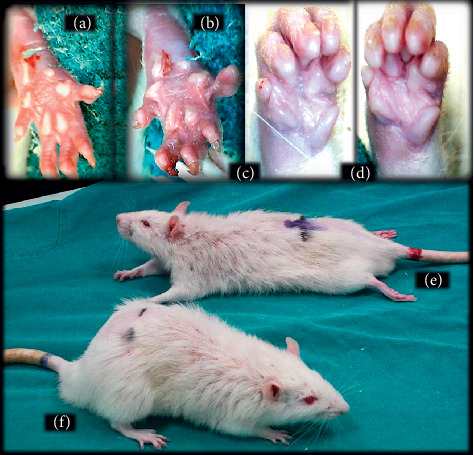
Illustrative presentation of the effects of the lidocaine-induced local anesthesia (intraplantar application (b) and axillar (d) and spinal (e) block) and corresponding BPC 157 counteraction ((a), (c), and (f)). Hind limb, plantar, and digits oedema in control rats (b) and counteraction in BPC 157 rats (a) immediately after 120 sec hot plate test. Fore limb, palmar, and fingers oedema in control rats (d) and counteraction in BPC 157 rats (c) presented at 30 min following lidocaine-induced axillary block. Presentation of the intrathecal anesthesia effect: prolonged hind limbs function failure and long-lasting flaccid paralysis (e) and recovery in BPC 157 rats (f) at 50 min following lidocaine.

Intrathecal anesthesia produced prolonged hind limb function failure; long-lasting flaccid paralysis with full motor recovery (score 3) presented after 90 minutes of lidocaine-induced anesthesia (Figures 8–11). As seen in the counteracting of the axillary block effects, BPC 157 medication considerably counteracted the lidocaine-induced course, given immediately after lidocaine (with full motor recovery after 40 (*μ*g) or 50 minutes (*μ*g) of lidocaine application), or later (with full motor recovery after 45 (*μ*g) or 65 minutes (*μ*g) of lidocaine application). L-NAME, prophylactically or therapeutically, exhibited a consistent counteracting effect (with full recovery motoric function presented approximately 70 minutes after lidocaine application). L-arginine exhibited a distinctive effect, counteraction, when given immediately after lidocaine (similar to the L-NAME group), but prolongation (with full motoric function after 110 minutes of observation time) when given later. As in the case of the axillary block, given together prophylactically or therapeutically, L-NAME and L-arginine (L-NAME + L-arginine) counteracted the other's response with results similar to those of the control group. Likewise, BPC 157 maintained its original response when given together with L-NAME or L-arginine. When BPC 157 was given together with L-NAME and L-arginine, its original response reappeared (Figures 8–11).

#### 3.2.4. Seizures

Regularly, after lidocaine 80 mg/kg intraperitoneally, all rats exhibited severe tonic-clonic convulsions, unless BPC 157 10 *μ*g/kg, 10 ng/kg intraperitoneally, was given immediately after lidocaine. Of note, none of the lidocaine-treated rats that received BPC 157 therapy had convulsions and they all maintained undisturbed normal behaviour (*P* < 0.05 vs. control). Otherwise, the convulsions in lidocaine-treated rats started within a minute of the application, i.e., 8 ± 1 minutes, and lasted for the next 40 ± 2 minutes before the rats' recovery.

### 3.3. The Effect of BPC 157 on Lidocaine-Induced Depolarisation of HEK293 Cells

Lidocaine (1 mM) and BPC 157 (1 *μ*m) were dissolved in a Ringer's-type solution. The effects of tested substrates were calculated by comparing the membrane potential measured during substrate application to the cell and the membrane potential measured while perfusing the cells with Ringer's-type solution alone. In the presence of 1 *μ*m BPC 157, lidocaine-induced depolarisation was inhibited.

## 4. Discussion

We demonstrated that the stable gastric pentadecapeptide BPC 157 [1–13] strongly counteracts the local anesthetic effect of lidocaine, infiltrative cutaneous analgesia, and axillary and spinal intrathecal blocks. This counteracting effect is related to the NO-system, as shown by BPC 157 application with NO-agents, the NOS-blocker L-NAME and the NOS-substrate L-arginine, given alone and/or together. Also, in the same dosage range, BPC 157 antagonises lidocaine-induced bradycardia. Likewise, BPC 157 counteracts lidocaine-induced convulsions. In addition to the lidocaine-induced arrhythmias, local anesthesia, and convulsions effects in rats, we also demonstrated a particular pentadecapeptide BPC 157-lidocaine interaction under in vitro conditions that BPC 157 counteracts lidocaine-induced depolarisation in nontransfected HEK293 cells.

All local anesthetics carry a dose-dependent risk of possible CNS and cardiovascular toxicity [46]. Therefore, the noted evidence (BPC 157 markedly interacts with lidocaine-induced adverse effects) combines and extends the previous demonstration that BPC 157 counteracts bupivacaine cardiotoxicity [14] and can therefore be a potential antidote to amide local anesthetics. The noted counteraction of the bupivacaine toxicity (14), much like that of lidocaine, is quite indicative. Namely, bupivacaine has greater potential for direct cardiac toxicity than other agents, and thereby, a greater affinity for the inactive and resting sodium channel configurations and slower dissociation from these channels [46]. All together, these findings support an even more effective counteraction of all lidocaine-induced adverse effects, as noted here. As bupivacaine administration induced characteristic severe cardiac disturbances (bradycardia, prolongation of all of the observed waves and intervals, AV block, ventricular ectopy, ventricular tachycardia, T-wave elevation, and asystole) [14], the cardiac effects, largely counteracted by the administration of BPC 157 [14], may support the therapy principle and beneficial effects observed in the lidocaine-treated rats [46]. Consequently, there may be a difference between cardiac and local anesthetic lidocaine effects (the outward positioning of the DIII and DIV S4s necessary to achieve the high affinity configuration for neuronal sodium channel isoforms) [47]; these findings strongly support the further counteracting effects of BPC 157 administration on lidocaine-induced infiltrative cutaneous analgesia and axillary and spinal intrathecal blocks. Moreover, these findings would approach the BPC 157 counteracting effect to the essential chain of the events following the application of local anesthetic [46, 47]. This may be essential for the penetration of both the epineurium and neuronal membrane, during the action, for the time that a local anesthetic remains in close proximity to neural fibres, since lidocaine shortens its own duration by dilating local vasculature [46]. Providing that the local sequestration of highly lipid-soluble anesthetics may allow for continual release to the neuronal membranes, prolonging duration [46], it is important that BPC 157 administration-induced counteraction accordingly occurs with the immediate application after lidocaine, as well as when given later after lidocaine, with a 10-minute delay. Thus, BPC 157 may interfere with the early events, as well as with the already established late lidocaine-induced effects. BPC 157 administration may both attenuate the development of lidocaine-induced adverse effects and reverse the preexisting local anesthetic effects of lidocaine.

As mentioned, BPC 157 largely interacts with NO-system in different models and species, with both L-NAME and L-arginine effects [1–13]. In axillary and spinal intrathecal blocks, an indicative point is that the counteracting effect of the BPC 157 administration occurs also in the presence of the NOS-blockade (L-NAME + BPC 157) as well as NOS-substrate application (L-arginine + BPC 157). Moreover, it occurs under the circumstances of the particular involvement of the NO-system (L-NAME and L-arginine have a parallel effect since both L-NAME and L-arginine, when given alone, shorten the duration of the lidocaine effect). When L-NAME and L-arginine are given together (L-NAME + L-arginine), they antagonise each other's counteracting effect (and the subsequent NO-system-specific effect). The distinctive effect of L-arginine when given later (prolongation of the lidocaine effect) may indicate particular NO-system involvement depending on particular distinctions in the early and late lidocaine course that may be distinctively affected by the application of NO-agents. However, in any circumstance, early or late lidocaine course, additional BPC 157 coapplication (L-NAME + L-arginine + BPC 157) would reestablish the counteraction over lidocaine-induced effect (thereby particular relations of BPC 157 with NO-system). Of note, a similar NO-system presentation (parallel effects of NOS-blockade (L-NAME) and NOS-stimulation (NOS-substrate L-arginine) that combined (L-NAME + L-arginine) antagonise each other) was also noted in other studies, with various models [48–52]. This provides a common model [48–52]; that particular NO-system involvement is also behind lidocaine-induced adverse effects. The inconsistent points about NO-system involvement, different L-NAME and/or different L-arginine effects, in various events, may be related to the facts that the NO-lidocaine studies rarely applied the used complete application of NO-agents (i.e., both L-NAME and L-arginine, given both alone and together) with the investigated procedure or agent application [53–56].

Commonly, convulsive seizures are the initial life-threatening consequence of local anesthetic overdose [46]. Thereby, the evidenced counteraction of the lidocaine-induced seizures should be considered as the final point in the BPC 157 counteracting relationship to the lidocaine-induced arrhythmias, local anesthesia, and convulsions effects. In this, the BPC 157 anticonvulsant effect should first consider lidocaine seizures as the selective depression of central inhibitory tracts, which allows excitatory tracts to run amuck [46].

However, the full pleiotropic complexity of the lidocaine-induced convulsion illustrates the various systems implicated (i.e., the NMDA and non-NMDA receptors systems, NO-system, serotonin, GABA, dopamine, and noradrenaline) [56–61]. There are various agents counteracting lidocaine (such as NMDA antagonists [58, 59], L-NAME [56], ivermectin [62], amitriptiline [63], lipid emulsion [64], alpha-methyl-p-tyrosine, disulfiram [61], emulsified isoflurane [65], and propranolol [66]). Likewise, there are various agents potentiating lidocaine-induced convulsions (such as L-arginine [56], reserpine, methysergide [57], reduction in PaCO₂ and/or increase in PaO₂ [67], 4L-DOPA, methamphetamine, desipramine [61], and ketamine) [65]. Thereby, it may be important that the BPC 157 anticonvulsant potential counteracts variously induced seizures in rats and mice [27, 37, 39, 69]. Also, BPC 157 interacts with several systems [1–13] implicated in lidocaine-induced convulsions [56–61] and may therefore modulate their activities. Illustrating its particular activities, it counteracts the effects of the application of neuroleptics [22], as well as the application of amphetamines [70]. Given peripherally, it induces the release of serotonin in particular brain areas (i.e., nigrostriatal), exhibits a prominent antidepressant effect, and counteracts serotonin syndrome in rats [71–73].

Therefore, the stable gastric pentadecapeptide BPC 157 can be quite a distinctive “healing antidote” for the adverse effects of lidocaine. Namely, BPC 157 produced analgesia in the MgSO_4_ and acetic acid intraperitoneal test [41], as it counteracts peritonitis in several models [48–50, 74]. It possibly counteracts lidocaine-induced infiltrative cutaneous analgesia due to its healing effect (thereby causing markedly less oedema and tissue damage after axillary block or after the hot plate test, as BPC 157 prominently heals even severe burns) [75–77]. Likewise, in rats with a severed sciatic nerve, BPC 157 markedly improves nerve healing and counteracts autotomy [35], which reflects chronic neuropathic pain, neuroma at the proximal nerve stump, and regenerative nerve sprouts growing into all directions, and prevents or at least significantly attenuates the chain of events otherwise leading to the painful sensation referred to the denervated region [78]. A corresponding autotomy counteraction appears in the L2-L3 spinal compression model, when the stable gastric pentadecapeptide BPC 157 improves the healing course of the spinal cord injury and leads to functional recovery in rats [79]. Also, much like lidocaine-induced arrhythmias, BPC 157 counteracts disturbances after bupivacaine [14], KCl overdose [15], digitalis overdose [16], hypoxia and reoxygenation [17], succinylcholine [18] neuroleptics and prokinetics [19], and doxorubicin [80]. As mentioned, much like lidocaine-induced adverse effects, BPC 157 counteracts morphine-induced analgesia [26] and counteracts NSAID-induced toxicity [7, 24, 27–34].

Also, BPC 157 may have a particular effect on vasculature that could influence the duration of the lidocaine effect (i.e., lidocaine shortens its own duration by dilating local vasculature) [46]. This may be the rapid activation of the bypassing loop that occurs in the rat with the infrarenal occlusion of the inferior caval vein (and thereby resolved Virchow) much like in the rats with ischemic/reperfusion colitis, duodenal venous congestion lesions, a perforated caecum, bile duct ligation-induced liver cirrhosis, and portal hypertension [48–50, 74, 81]. Accordingly, BPC 157 interacts with several molecular pathways [1, 74, 82–88]. In particular, BPC 157 increased the expression and internalisation of VEGFR2 and the activation of the VEGFR2-Akt-eNOS signalling pathway without the need for other known ligands or shear stress [85].

Finally, in vitro inhibition of the depolarisation of HEK293 cells by lidocaine fully supports the hypothesis that BPC 157 may, with a direct action, successfully counteract the effect of lidocaine much like the effect of bupivacaine [14]. Likewise, cell depolarisation due to increasing magnesium concentrations was inhibited in the presence of BPC 157 (1 *μ*M) in vitro [51]. Furthermore, BPC 157 reduces the depolarisation caused by hyperkalaemia in HEK293 cells [15]. The depolarisation caused by BPC 157 (1 *μ*m) was inhibited by the application of the nonspecific potassium blocker BaCl_2_ (1 mM) [15]. Thus, to counteract lidocaine-induced disturbances, as well as local anesthetic-induced disturbances, BPC 157 may have an effect of its own on membrane potential.

## 5. Conclusions

In conclusion, we suggest BPC 157 as the antidote for local anesthetics, in particular for the lidocaine-induced arrhythmias, infiltrative cutaneous analgesia, axillary and spinal intrathecal blocks, and seizures.

## Figures and Tables

**Figure 12:**
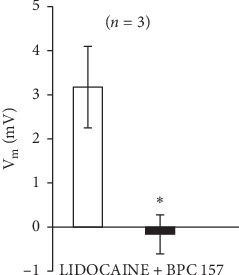
Lidocaine (1 mM) depolarized HEK293 cells which was inhibited in the presence of 1 *μ*M BPC 157. Results are presented as average ± SEM. ^*∗*^*P* < 0.05 statistically significant difference compared to effects of lidocaine.

## Data Availability

The datasets used and/or analysed during the current study are available from the corresponding author upon reasonable request.
